# Mitochondrial non-coding RNAs as novel biomarkers and therapeutic targets in lung cancer integration of traditional bioinformatics and machine learning approaches

**DOI:** 10.3389/fonc.2025.1690077

**Published:** 2025-10-30

**Authors:** Liu Haoming, Wang Rui, Hua Mao, Jiang Fan, Zhang Li, Sun Xin, Ren Hong

**Affiliations:** ^1^ Department of Thoracic Surgery, The First Affiliated Hospital of Xi ‘an Jiaotong University, Xi’an, Shaanxi, China; ^2^ Department of Breast, Qinghai Fifth People’s Hospital, Xining, Qinghai, China; ^3^ Zhejiang Cancer Hospital, Hangzhou Institute of Medicine (HIM), Chinese Academy of Sciences, Hangzhou, Zhejiang, China; ^4^ Department of Radiotherapy, Qinghai Fifth People’s Hospital, Xining, Qinghai, China; ^5^ Department of Orthopedics, Qinghai Fifth People’s Hospital, Xining, Qinghai, China; ^6^ Department of Otolaryngology - Thyroid Gland Surgery, Qinghai Fifth People’s Hospital, Xining, Qinghai, China

**Keywords:** lung cancer, machine learning, biomarker discovery, mitochondrial non-coding RNAs, potential drug targets

## Abstract

**Background:**

Lung cancer diagnosis requires cost-effective biomarkers. Mitochondrial non-coding RNAs (mtRNAs) represent unexplored diagnostic targets.

**Methods:**

We analyzed TCGA-LUAD/LUSC miRNA-seq data to identify mtRNAs via mitochondrial genome alignment. Machine learning algorithms (SVM, Random Forest, Logistic Regression) classified samples using differentially expressed mtRNAs (P < 0.01, |log2FC| > 1). Top-ranked t00043332 was functionally validated in A549/PC9 cells.

**Results:**

Ten mtRNAs distinguished cancer from normal tissues. Random Forest and Logistic Regression achieved superior classification (AUC > 0.92) versus SVM. Nine mtRNAs were upregulated, one downregulated in cancer. No survival associations were observed. t00043332 overexpression promoted proliferation, migration, invasion, and apoptosis resistance.

**Conclusion:**

mtRNAs serve as effective lung cancer diagnostic biomarkers through integrated traditional and AI approaches. t00043332 functions as an oncogene, providing therapeutic targets and advancing biomarker discovery.

## Introduction

Lung cancer remains the leading cause of cancer-related mortality worldwide, accounting for approximately 1.8 million deaths annually and representing nearly 20% of all cancer deaths (1, 2). Despite significant advances in therapeutic interventions and diagnostic technologies over the past decades, the five-year survival rate for lung cancer patients remains disappointingly low at approximately 15-20%, primarily due to late-stage diagnosis when curative treatments are no longer viable (3). The heterogeneous nature of lung cancer, encompassing multiple histological subtypes including adenocarcinoma, squamous cell carcinoma, and small cell lung carcinoma, further complicates early detection and therapeutic management (4, 5). Current screening methodologies, particularly low-dose computed tomography (LDCT), have demonstrated efficacy in reducing mortality rates by enabling earlier detection in high-risk populations (6). However, these imaging-based approaches are associated with substantial limitations including high costs, limited accessibility, frequent false-positive results leading to unnecessary invasive procedures, and radiation exposure concerns that restrict their widespread implementation as population-based screening tools (7, 8).

The urgent need for cost-effective, minimally invasive, and highly accurate diagnostic biomarkers has driven extensive research into liquid biopsy approaches, particularly focusing on circulating nucleic acids in peripheral blood (9, 10). Small non-coding RNAs (sncRNAs) have emerged as promising biomarker candidates due to their remarkable stability in circulation, tissue-specific expression patterns, and functional roles in cancer pathogenesis (11, 12). While microRNAs (miRNAs) have been extensively studied as diagnostic and prognostic biomarkers in various cancer types (13), recent investigations have expanded to include other classes of sncRNAs such as small nucleolar RNAs (snoRNAs) (14), PIWI-interacting RNAs (piRNAs) (15), and transfer RNA-derived small RNAs (tsRNAs) (16). However, one relatively unexplored class of regulatory sncRNAs with significant potential for cancer biomarker discovery is mitochondria-derived small RNAs (mtRNAs), which represent a novel category of non-coding RNAs generated from mitochondrial tRNA precursors through specific cleavage mechanisms (17).

Mitochondria play fundamental roles in cellular energy metabolism, apoptosis regulation, and oxidative stress responses, all of which are critically disrupted during cancer development and progression (18, 19). The human mitochondrial genome contains 37 genes encoding 13 protein-coding genes, 22 transfer RNAs, and 2 ribosomal RNAs, all of which are essential for mitochondrial respiratory chain function and ATP synthesis (20, 21). Emerging evidence suggests that mitochondrial dysfunction, characterized by altered metabolism, increased reactive oxygen species production, and compromised respiratory chain activity, represents a hallmark of cancer pathophysiology (22–24). Recent discoveries have revealed that mitochondrial tRNAs undergo specific endonucleolytic cleavage to generate stable small RNA fragments, termed mtRNAs, which exhibit tissue-specific expression patterns and potential regulatory functions analogous to cytoplasmic miRNAs (25, 26). These mtRNAs have been implicated in various cellular processes including stress responses, metabolic regulation, and potentially cancer development, yet their diagnostic utility in human malignancies remains largely unexplored (27, 28).

The integration of artificial intelligence and machine learning approaches with traditional biomarker discovery methodologies offers unprecedented opportunities to enhance the accuracy and reliability of cancer diagnostic tools (29). Machine learning algorithms, particularly ensemble methods such as Random Forest, Support Vector Machines, and deep learning architectures, have demonstrated superior performance in handling high-dimensional genomic data and identifying complex biomarker signatures that may not be apparent through conventional statistical approaches (30, 31). These computational methods can effectively manage the challenges associated with small sample sizes, high-dimensional feature spaces, and the need for robust cross-validation, making them ideally suited for biomarker discovery applications (32). Furthermore, the development of ratio-based normalization methods has addressed longstanding challenges in sncRNA biomarker studies, particularly the issues related to technical variability, batch effects, and the absence of reliable reference genes for normalization (17).

In this study, we present a comprehensive investigation combining traditional bioinformatics approaches with advanced machine learning methodologies to identify and validate mtRNA biomarkers for lung cancer diagnosis. Through systematic analysis of TCGA datasets encompassing both lung adenocarcinoma and squamous cell carcinoma, we aimed to characterize the mtRNA expression landscape in lung cancer, develop robust machine learning-based classification models, and validate the functional significance of identified biomarkers through *in vitro* experimental approaches. Our integrated strategy represents a novel application of both traditional molecular biology techniques and artificial intelligence approaches to advance the discovery of therapeutically relevant biomarkers and elucidate novel therapeutic mechanisms in lung cancer pathogenesis.

## Methods

### Data acquisition and processing

The miRNA-seq data from The Cancer Genome Atlas (TCGA) database were obtained for lung adenocarcinoma (TCGA-LUAD, n=513) and lung squamous cell carcinoma (TCGA-LUSC, n=478) cohorts with their corresponding normal tissue samples (**Supplementary Table S1**). Raw sequencing data in BAM format were retrieved from the TCGA data portal and processed using standardized bioinformatics pipelines. Quality control assessment was performed using FastQC (Babraham Bioinformatics, Cambridge, UK) to evaluate read quality, adapter contamination, and sequence length distribution. Adapter sequences were trimmed using Cutadapt version 3.4 (Marcel Martin, TU Dortmund University, Germany) with stringent parameters to ensure high-quality reads for downstream analysis.

### Mitochondrial RNA identification and quantification

Mitochondrial non-coding RNAs (mtRNAs) were identified and quantified using the methodology described by Yu et al. (2023) (17). Briefly, trimmed sequencing reads were aligned to the human mitochondrial genome (NC_012920.1) using STAR aligner version 2.7.9a (Cold Spring Harbor Laboratory, NY, USA) with optimized parameters for small RNA alignment. The mitochondrial tRNA database (MitotRNAdb) was used as the reference for mtRNA annotation and classification. Read counts for each mtRNA were obtained using HTSeq-count from the HTSeq package version 0.13.5 (European Molecular Biology Laboratory, Heidelberg, Germany) with intersection-strict mode to ensure accurate quantification. Raw count matrices were normalized using the trimmed mean of M-values (TMM) method implemented in the edgeR package version 3.38.4 (Bioconductor, Fred Hutchinson Cancer Research Center, Seattle, WA, USA).

### Differential expression analysis and statistical testing

Differential expression analysis was conducted to identify significantly altered mtRNAs between tumor and adjacent normal tissue samples. Paired sample analysis was performed using the limma package version 3.52.4 (Bioconductor) for linear modeling of gene expression data. Statistical significance was determined using empirical Bayes moderated t-statistics with Benjamini-Hochberg false discovery rate (FDR) correction for multiple testing. mtRNAs with adjusted p-values < 0.01 and absolute log2 fold change > 1 were considered significantly differentially expressed. Principal component analysis (PCA) was performed using the prcomp function in R version 4.2.0 (R Foundation for Statistical Computing, Vienna, Austria) to visualize sample clustering patterns based on significantly dysregulated mtRNAs.

### Machine learning classification and feature selection

Multiple machine learning algorithms were employed to evaluate the diagnostic potential of mtRNAs and identify optimal feature combinations. Support Vector Machine (SVM) classification was implemented using the e1071 package version 1.7-11 (Vienna University of Technology, Austria) with radial basis function kernel and optimized hyperparameters determined through 10-fold cross-validation. Random Forest classification was performed using the randomForest package version 4.7-1.1 (University of California, Berkeley, CA, USA) with 1000 trees and mtry parameter optimized for classification tasks. Logistic regression modeling was conducted using the glm function in base R with binomial family specification. Model performance was evaluated using receiver operating characteristic (ROC) curve analysis implemented in the pROC package version 1.18.0 (University of Geneva, Switzerland). Feature importance ranking was determined using the Random Forest variable importance measures, specifically mean decrease in accuracy and mean decrease in Gini impurity.

### Cell culture and transfection

Human lung adenocarcinoma cell lines A549 (ATCC CCL-185) and PC9 (RIKEN BioResource Research Center, Japan) were cultured in RPMI-1640 medium (Gibco, Thermo Fisher Scientific, Waltham, MA, USA) supplemented with 10% fetal bovine serum (FBS, Gibco), 100 U/mL penicillin, and 100 μg/mL streptomycin (Gibco) at 37 °C in a humidified atmosphere with 5% CO2. Cells were routinely tested for mycoplasma contamination using the MycoAlert Mycoplasma Detection Kit (Lonza, Basel, Switzerland). For overexpression experiments, custom mtRNA mimics targeting t00043332 and negative control oligonucleotides were synthesized by GenePharma Co., Ltd. (Shanghai, China). Transfections were performed using Lipofectamine 3000 reagent (Invitrogen, Thermo Fisher Scientific) according to the manufacturer’s protocol with a final oligonucleotide concentration of 50 nM.

### Cell proliferation and viability assays

Cell proliferation was assessed using the Cell Counting Kit-8 (CCK-8, Dojindo Molecular Technologies, Kumamoto, Japan) colorimetric assay. Cells were seeded in 96-well plates at a density of 3,000 cells per well and transfected with mtRNA mimics or negative controls. At designated time points (24, 48, 72, and 96 hours post-transfection), 10 μL of CCK-8 solution was added to each well and incubated for 2 hours at 37 °C. Absorbance was measured at 450 nm using a microplate reader (BioTek Instruments, Winooski, VT, USA). Colony formation assays were performed by seeding 500 transfected cells per well in 6-well plates and culturing for 14 days. Colonies were fixed with 4% paraformaldehyde (Sigma-Aldrich, St. Louis, MO, USA) and stained with 0.5% crystal violet solution (Beyotime Biotechnology, Shanghai, China). Colonies containing more than 50 cells were manually counted under light microscopy.

### Cell migration and invasion assays

Cell migration capacity was evaluated using wound healing scratch assays. Transfected cells were seeded in 6-well plates until reaching 90% confluence, followed by creation of standardized scratches using sterile pipette tips. Cells were then cultured in serum-free medium, and wound closure was monitored at 0- and 48-hours using phase-contrast microscopy (Olympus Corporation, Tokyo, Japan). Wound closure percentage was calculated using ImageJ software version 1.53t (National Institutes of Health, Bethesda, MD, USA). For invasion assays, Transwell chambers with 8-μm pore size polycarbonate membranes (Corning Inc., Corning, NY, USA) were coated with Matrigel (BD Biosciences, Franklin Lakes, NJ, USA) diluted 1:8 in serum-free medium. Transfected cells (2×10^4) in serum-free medium were added to the upper chamber, while the lower chamber contained complete medium with 10% FBS as a chemoattractant. After 24 hours of incubation, non-invading cells on the upper surface were removed, and invading cells on the lower surface were fixed with methanol and stained with 0.1% crystal violet. Invading cells were counted in five random fields per membrane under light microscopy.

### Flow cytometry analysis

Apoptosis detection was performed using the Annexin V-FITC/Propidium Iodide Apoptosis Detection Kit (BD Biosciences) according to the manufacturer’s instructions. Transfected cells were harvested 48 hours post-transfection, washed twice with cold phosphate-buffered saline (PBS, Gibco), and resuspended in binding buffer at a concentration of 1×10^6 cells/mL. Cells were stained with 5 μL Annexin V-FITC and 5 μL propidium iodide for 15 minutes at room temperature in the dark. Flow cytometric analysis was performed using a BD FACSCalibur flow cytometer (BD Biosciences) with data acquisition of 10,000 events per sample. Data analysis was conducted using FlowJo software version 10.8.1 (Becton, Dickinson and Company, Franklin Lakes, NJ, USA) to determine the percentage of viable, early apoptotic, late apoptotic, and necrotic cell populations.

### Statistical analysis

All statistical analyses were performed using R version 4.2.0 and GraphPad Prism version 9.0 (GraphPad Software, San Diego, CA, USA). Data are presented as mean ± standard error of the mean (SEM) from at least three independent experiments. Comparisons between two groups were analyzed using unpaired two-tailed Student’s t-tests for continuous variables. For survival analysis, patients were stratified into high and low expression groups based on median mtRNA expression levels, and overall survival was analyzed using Kaplan-Meier curves with log-rank tests. A p-value < 0.05 was considered statistically significant, and multiple testing correction was applied where appropriate using the Benjamini-Hochberg method.

## Results

### Differential expression analysis of mitochondrial non-coding RNAs in lung cancer

**Figure 1:**
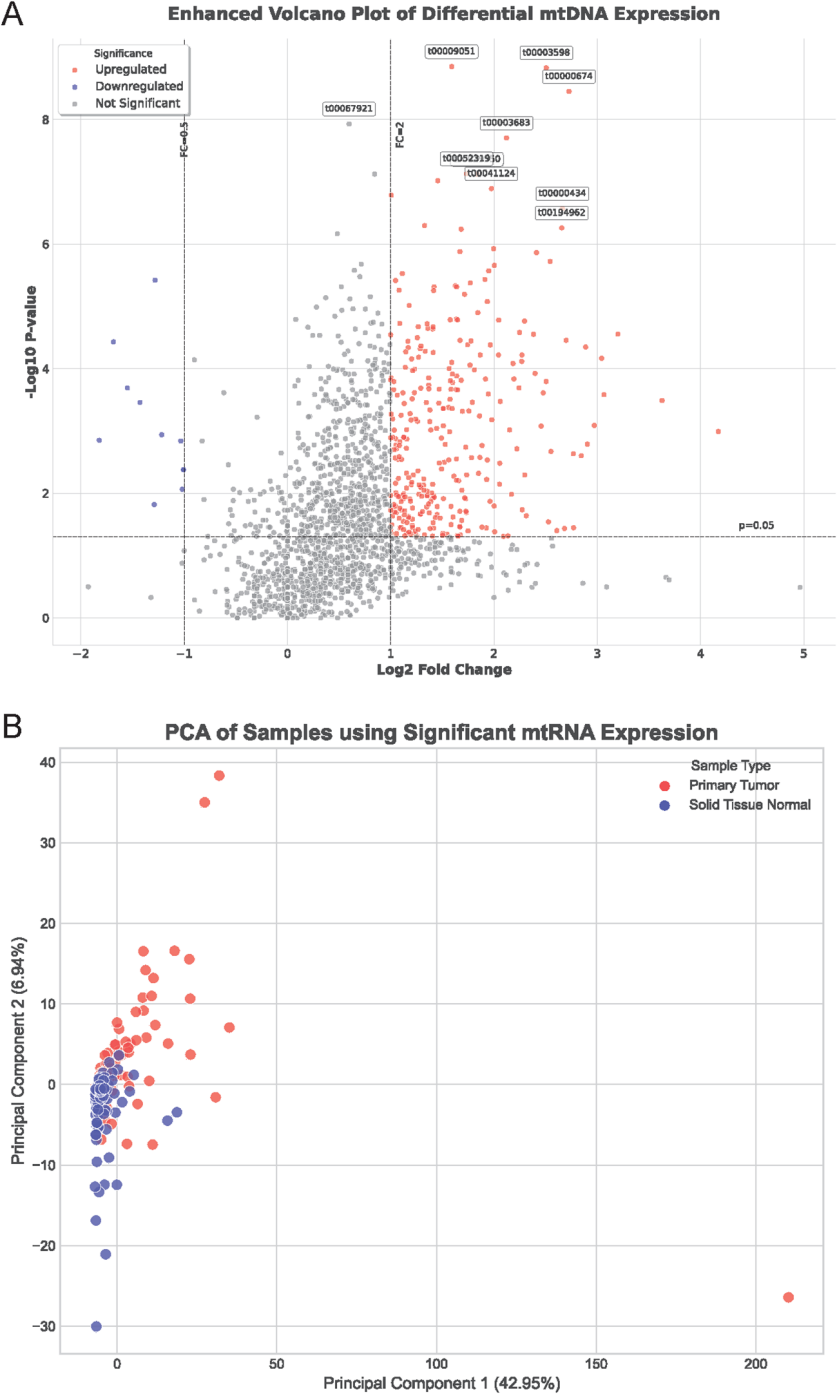
Differential expression analysis of mitochondrial non-coding RNAs in lung cancer. **(A)** Enhanced volcano plot displaying the distribution of mtRNAs based on fold change (x-axis) and statistical significance (-log10 P-value, y-axis) between lung cancer and normal tissue samples. Red dots represent significantly upregulated mtRNAs, blue dots indicate significantly downregulated mtRNAs, and gray dots show non-significant changes. Horizontal dashed line indicates P = 0.01 threshold, vertical dashed lines represent |log2FC| = 1 cutoffs. Selected mtRNAs with highest significance are labeled with their identifiers. **(B)** Principal Component Analysis (PCA) plot showing sample clustering based on significantly dysregulated mtRNA expression profiles. Red dots represent primary tumor samples; blue dots indicate solid tissue normal samples. PC1 explains 42.95% of total variance, PC2 explains 6.84% of total variance, demonstrating clear separation between cancer and normal tissue groups.

To investigate the role of mitochondrial non-coding RNAs (mtRNAs) in lung cancer pathogenesis, we performed comprehensive bioinformatics analysis using miRNA-seq data from The Cancer Genome Atlas (TCGA) database, specifically TCGA-LUAD and TCGA-LUSC datasets. Through alignment to the mitochondrial genome, we identified and quantified mtRNA expression levels in paired tumor and adjacent normal tissue samples. Differential expression analysis revealed significant alterations in mtRNA profiles between cancer and normal tissues. The enhanced volcano plot (**Figure 1A**) demonstrates the distribution of mtRNAs based on their fold change and statistical significance, with stringent criteria of P < 0.01 and |log2FC| > 1 applied to identify significantly dysregulated mtRNAs(**Supplementary Table S2**). Principal component analysis (PCA) of samples using significantly altered mtRNA expression profiles (**Figure 1B**) revealed distinct clustering patterns between primary tumor samples (red dots) and solid tissue normal samples (blue dots), indicating that mtRNA expression signatures can effectively discriminate between malignant and benign tissues with clear separation trends along the first principal component, which explained 42.95% of the total variance.

### Machine learning-based classification and feature selection of mtRNAs

**Figure 2:**
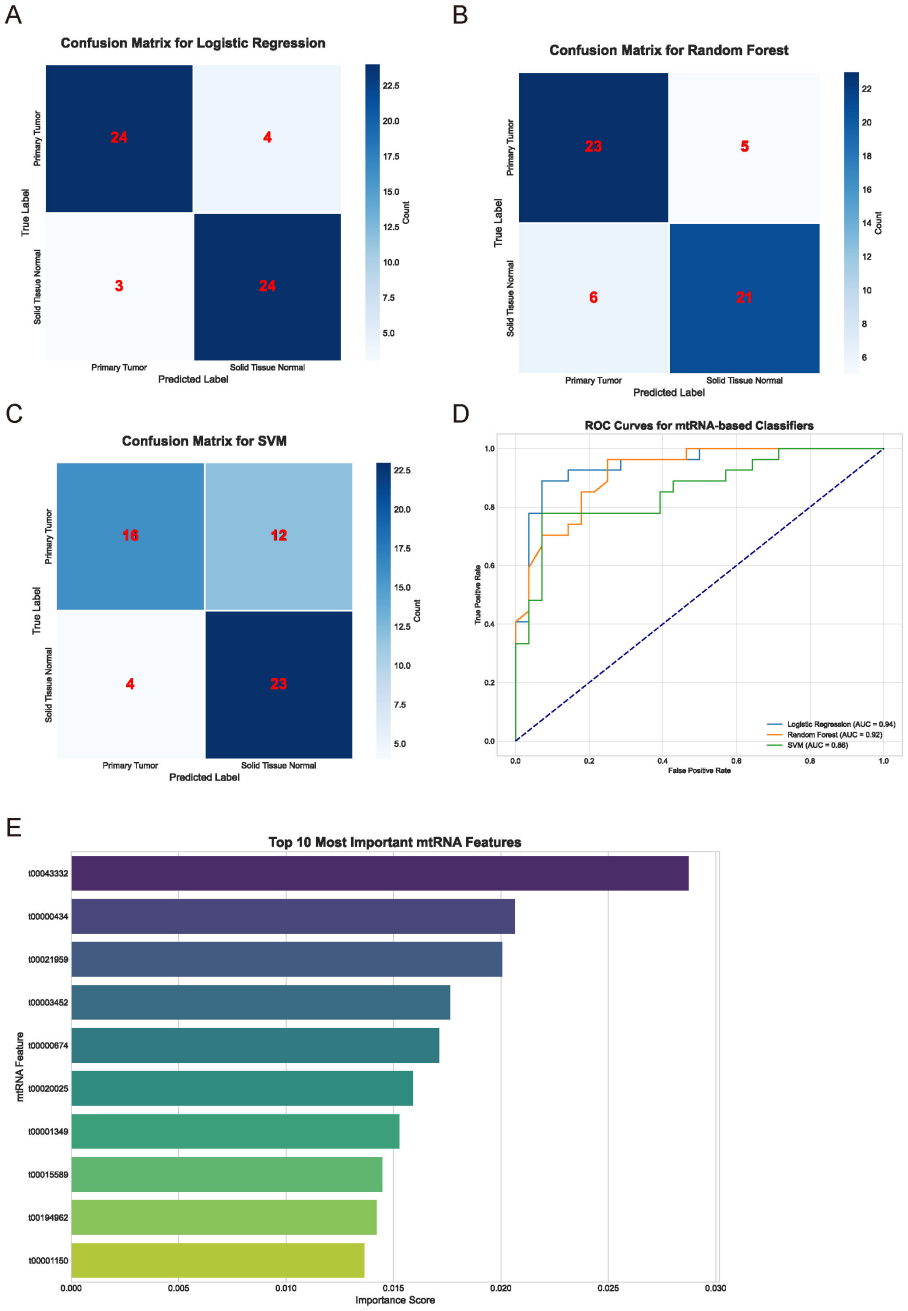
Machine learning-based classification performance and feature selection. **(A-C)** Confusion matrices displaying classification performance for Logistic Regression **(A)**, Random Forest **(B)**, and Support Vector Machine **(C)** algorithms. Numbers within matrices represent true positive, false positive, true negative, and false negative classifications for primary tumor versus solid tissue normal samples. Color intensity corresponds to classification frequency. **(D)** Receiver Operating Characteristic (ROC) curves comparing the three machine learning algorithms. Logistic Regression (AUC = 0.94), Random Forest (AUC = 0.92), and SVM performance are shown with corresponding area under curve values. Diagonal dashed line represents random classification baseline. **(E)** Feature importance ranking of the top 10 most discriminative mtRNAs identified by Random Forest algorithm. Horizontal bar chart displays importance scores (x-axis) for each mtRNA identifier (y-axis), with t00043332 showing the highest importance score followed by t00000434 and t00021959.

To evaluate the diagnostic potential of mtRNAs and identify the most discriminative features, we employed multiple machine learning algorithms including Support Vector Machine (SVM), Random Forest (RF), and Logistic Regression. The performance evaluation through confusion matrices (**Figures 2A-C**) demonstrated varying classification accuracies across different algorithms. The Random Forest classifier achieved superior performance with 23 true positives and 5 false negatives for primary tumors, while the Logistic Regression model showed comparable results with 24 true positives and 4 false negatives. The SVM classifier exhibited the poorest performance with 16 true positives and 12 false negatives. Receiver Operating Characteristic (ROC) curve analysis (**Figure 2D**) confirmed these findings, with Random Forest (AUC = 0.92) and Logistic Regression (AUC = 0.94) demonstrating nearly equivalent and superior performance compared to SVM. Feature importance analysis using the Random Forest algorithm identified the top 10 most significant mtRNAs contributing to cancer classification (**Figure 2E**), with t00043332 emerging as the most influential biomarker with the highest importance score, followed by t00000434 and t00021959.

### Expression patterns and clinical significance of top-ranked mtRNAs

**Figure 3:**
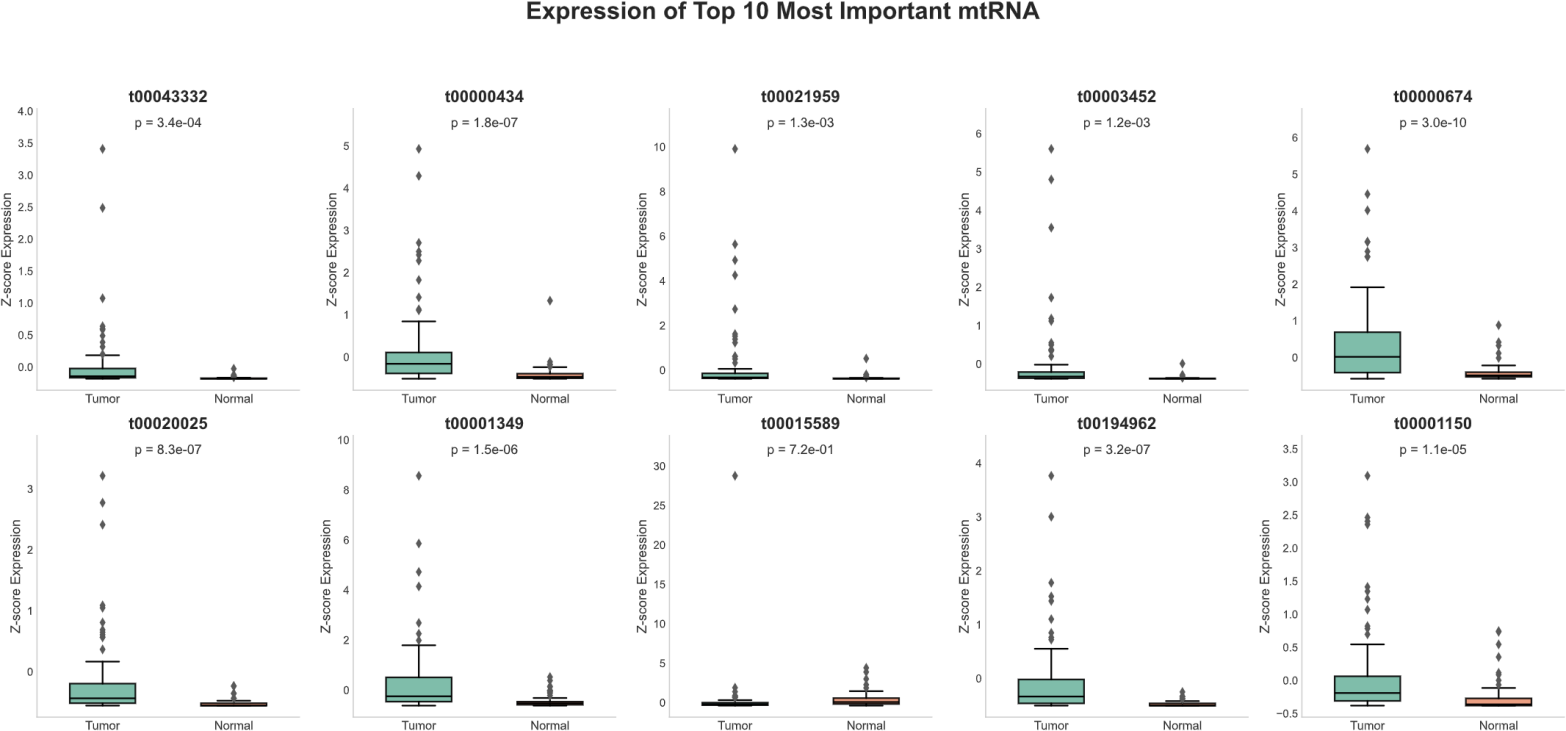
Expression profiles of top-ranked mitochondrial RNAs. Box plots showing normalized expression levels (Z-score) of the ten most important mtRNAs between tumor samples (n=91) and normal tissue samples (n=91). Each panel displays the mtRNA identifier, corresponding P-value, and sample size distribution. Box plots show median (center line), interquartile range (box), whiskers extending to 1.5× IQR, and individual data points as dots. Nine mtRNAs (t00043332, t00000434, t00021959, t00003452, t00000674, t00020025, t00001349, t00194962, t00001150) demonstrate significant upregulation in tumor samples, while t00015589 shows non-significant expression changes. Statistical significance was determined by paired t-test with P-values ranging from 1.1e-05 to 7.2e-01.

**Figure 4:**
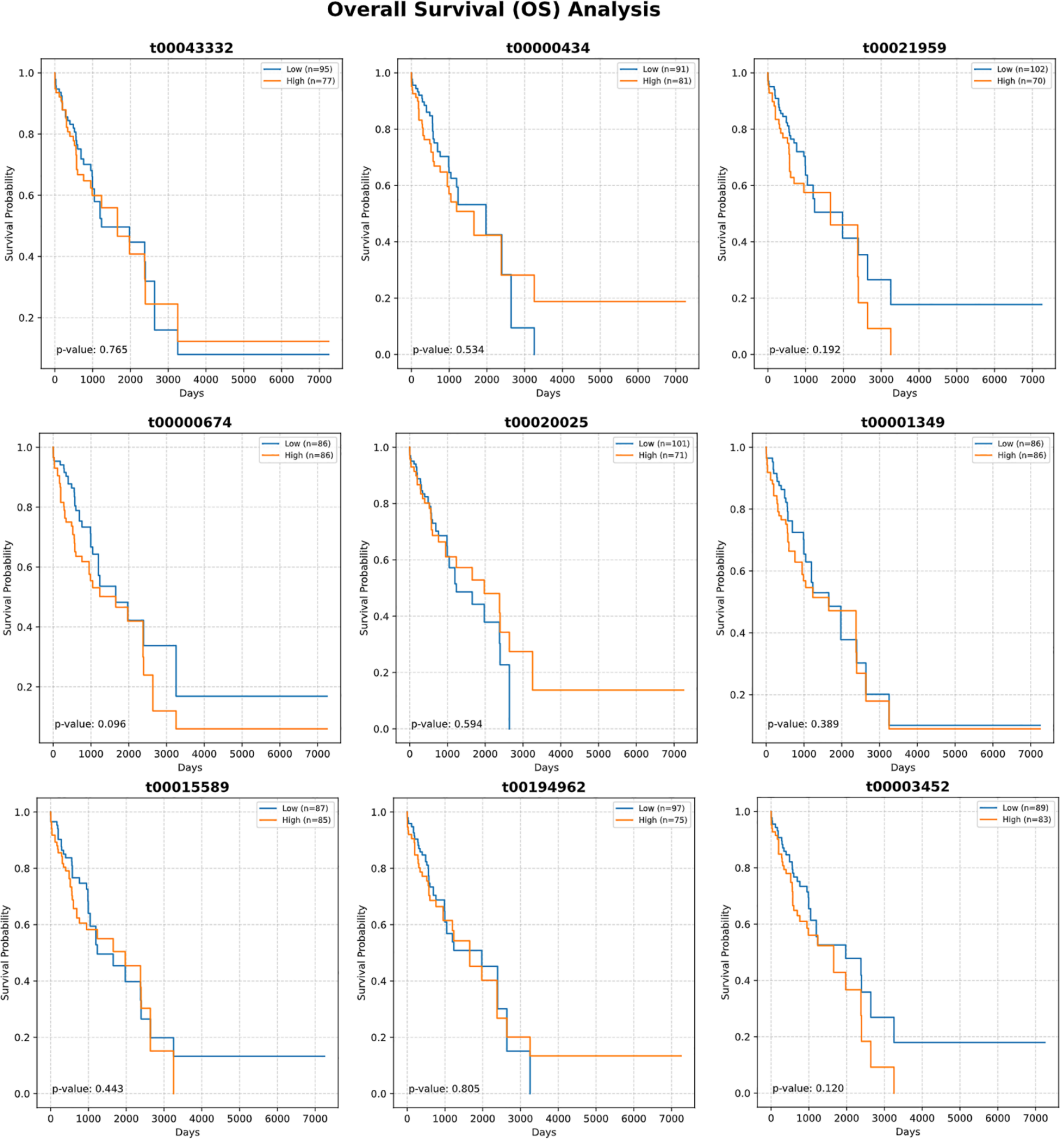
Survival analysis of top-ranked mitochondrial RNAs. Kaplan-Meier survival curves showing overall survival (OS) analysis for the ten most important mtRNAs. Each panel displays survival probability (y-axis) versus time in days (x-axis) for patients stratified into high (orange) and low (blue) expression groups based on median expression values. Sample sizes for each group are indicated in the legend. Log-rank test P-values are shown for each mtRNA, ranging from 0.096 to 0.805, indicating no statistically significant associations between mtRNA expression levels and patient survival outcomes. Survival curves demonstrate overlapping patterns between high and low expression groups across all analyzed mtRNAs.

Detailed expression analysis of the top 10 mtRNAs revealed distinct expression patterns between tumor and normal tissues (**Figure 3**). Among these biomarkers, nine mtRNAs including t00043332 (p = 3.4e-04), t00000434 (p = 1.8e-07), t00021959 (p = 1.3e-03), t00003452 (p = 1.2e-03), t00000674 (p = 3.0e-10), t00020025 (p = 8.3e-07), t00001349 (p = 1.5e-06), t00194962 (p = 3.2e-07), and t00001150 (p = 1.1e-05) showed significant upregulation in tumor tissues compared to normal tissues. Notably, t00015589 was the only mtRNA demonstrating significant downregulation in cancer tissues, though with marginal statistical significance (p = 7.2e-01). The expression levels were normalized as Z-scores, revealing substantial fold changes ranging from 2 to 8-fold increases in tumor samples. Survival analysis using the Kaplan-Meier method for overall survival (OS) was performed for all top 10 mtRNAs (**Figure 4**), stratifying patients into high and low expression groups based on median expression values. Interestingly, none of the mtRNAs demonstrated statistically significant associations with patient overall survival outcomes, with p-values ranging from 0.096 to 0.805, suggesting that while these mtRNAs serve as excellent diagnostic biomarkers, their prognostic value may be limited in this cohort.

### Functional validation of t00043332 in lung cancer cell lines

**Figure 5:**
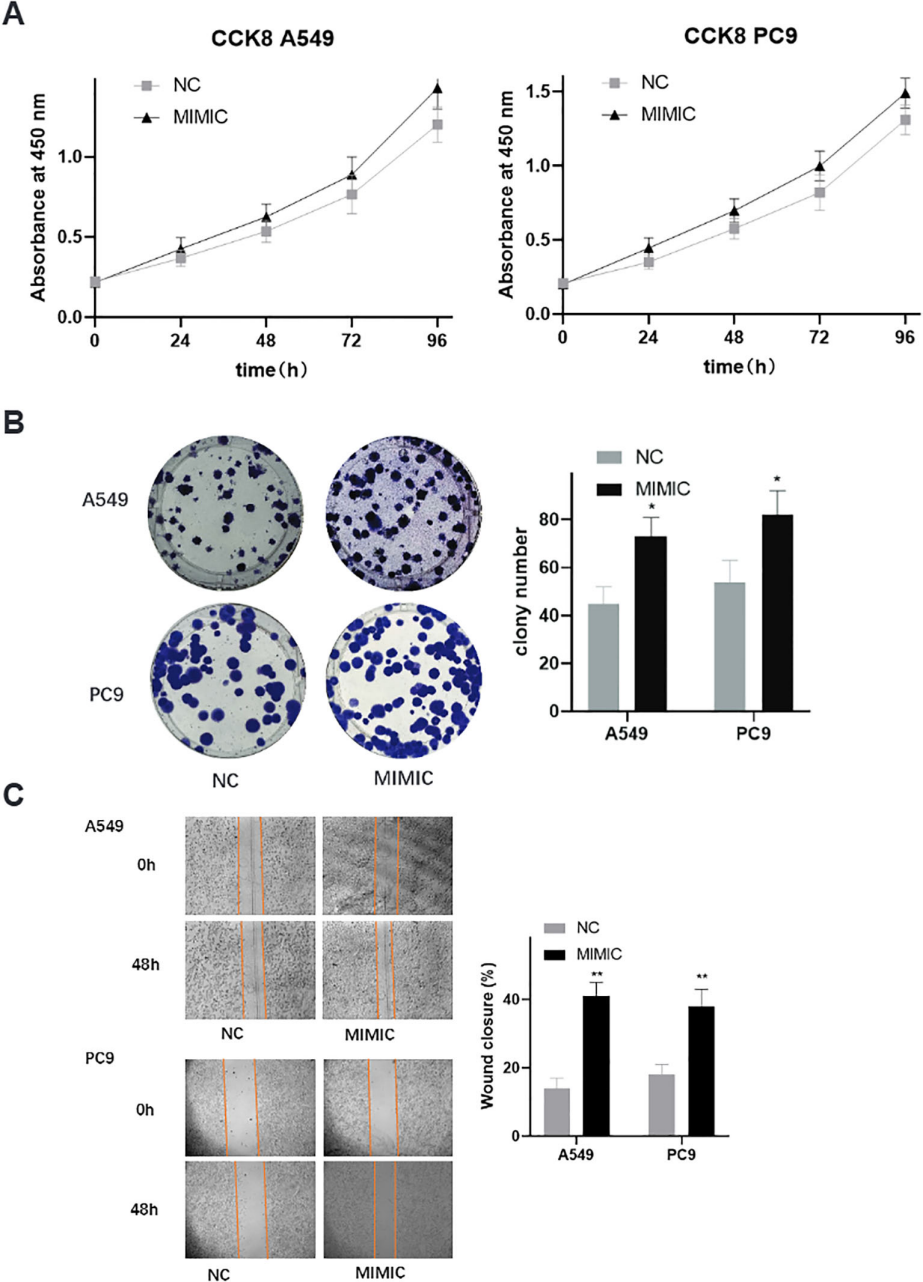
Functional validation of t00043332 in lung cancer cell lines - proliferation and migration. **(A)** Cell proliferation analysis using CCK-8 assays in A549 and PC9 cell lines over 96 hours. Gray squares represent negative control (NC), black triangles represent t00043332 mimic (MIMIC) treatment. Y-axis shows absorbance at 450 nm, x-axis shows time points. Error bars represent standard error from three independent experiments. Overexpression of t00043332 significantly promoted cell growth in both cell lines at all time points. **(B)** Colony formation assays showing representative colony images and quantification. Left panels show crystal violet-stained colonies for A549 (top) and PC9 (bottom) cells under NC and MIMIC conditions. Right panel shows colony number quantification with gray bars (NC) and black bars (MIMIC). Asterisk indicates P < 0.05 by t-test. **(C)** Wound healing assays demonstrating cell migration capacity. Left panels show representative microscopic images of scratch wounds at 0 and 48 hours for A549 (top) and PC9 (bottom) cells. Orange lines mark wound boundaries. Right panel shows wound closure percentage quantification with statistical significance indicated by asterisks (** P < 0.01).

To investigate the biological significance of the most prominent mtRNA biomarker, we performed functional studies by overexpressing t00043332 in two lung cancer cell lines, A549 (lung adenocarcinoma) and PC9 (lung adenocarcinoma with EGFR mutation). Cell proliferation assays using CCK-8 demonstrated that overexpression of t00043332 (MIMIC group) significantly promoted cell growth compared to negative control (NC) in both cell lines over a 96-hour time course (**Figure 5A**). The growth-promoting effect was consistently observed at all time points, with the most pronounced differences observed at 72 and 96 hours. Colony formation assays revealed that t00043332 overexpression substantially increased clonogenic capacity in both A549 and PC9 cells (**Figure 5B**), with colony numbers increasing from approximately 45 to 75 colonies in A549 cells and from 55 to 80 colonies in PC9 cells, representing statistically significant enhancements in cellular proliferation potential. Wound healing assays demonstrated enhanced migratory capacity in t00043332-overexpressing cells (**Figure 5C**), with wound closure percentages significantly increased in both cell lines (approximately 40% wound closure in MIMIC groups compared to 15-18% in control groups) after 48 hours, indicating accelerated cell migration and potentially enhanced metastatic potential.

### Enhanced invasive capacity and apoptosis resistance in t00043332-overexpressing cells

**Figure 6:**
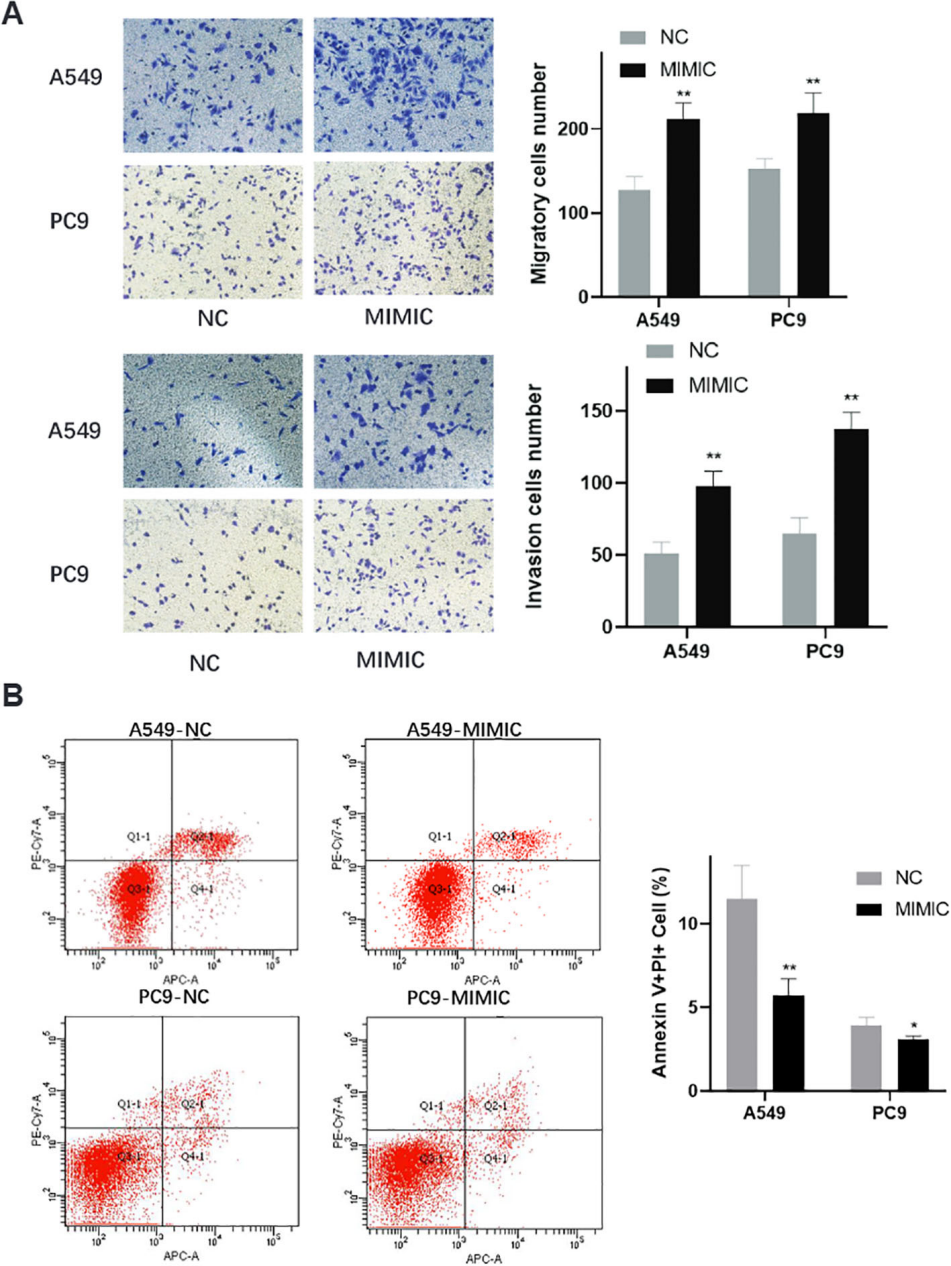
Enhanced invasive capacity and apoptosis resistance in t00043332-overexpressing cells. **(A)** Transwell invasion assays showing cell invasive capacity through Matrigel-coated membranes. Left panels display representative microscopic images of crystal violet-stained invading cells for A549 (top) and PC9 (bottom) under NC and MIMIC conditions. Right panels show quantification of invading cell numbers with gray bars (NC) and black bars (MIMIC) for migration (top) and invasion (bottom) assays. Statistical significance indicated by ** P < 0.01. **(B)** Flow cytometry analysis of apoptosis using Annexin V and propidium iodide staining. Left panels show representative flow cytometry scatter plots for A549-NC, A549-MIMIC, PC9-NC, and PC9-MIMIC conditions. Quadrants indicate live cells (Q3), early apoptotic cells (Q4), late apoptotic cells (Q2), and necrotic cells (Q1). Right panel shows quantification of Annexin V-positive cell percentages with gray bars (NC) and black bars (MIMIC). Statistical significance indicated by * P < 0.05 and ** P < 0.01, demonstrating reduced apoptosis in t00043332-overexpressing cells.

Transwell invasion assays provided additional evidence for the oncogenic role of t00043332 (**Figure 6A**). Overexpression of t00043332 significantly increased the number of invading cells through Matrigel-coated membranes in both A549 and PC9 cell lines. In A549 cells, the number of invading cells increased from approximately 125 to 210 cells per field, while in PC9 cells, invasion increased from about 150 to 220 cells per field, demonstrating enhanced invasive capabilities that could contribute to metastatic progression. Flow cytometry analysis using Annexin V and propidium iodide staining was performed to assess the effect of t00043332 on apoptosis (**Figure 6B**). The results revealed that overexpression of t00043332 led to a significant reduction in apoptotic cell populations. In A549 cells, the percentage of Annexin V-positive cells decreased from approximately 12% in the control group to 6% in the MIMIC group, while in PC9 cells, apoptosis decreased from about 4% to 3%. These findings suggest that t00043332 not only promotes proliferation and invasion but also confers resistance to programmed cell death, thereby contributing to tumor progression through multiple oncogenic mechanisms.

## Discussion

Our comprehensive investigation represents the first systematic characterization of mitochondrial non-coding RNAs as diagnostic biomarkers in lung cancer, demonstrating the successful integration of traditional bioinformatics methodologies with advanced machine learning approaches to identify novel therapeutic targets and biomarkers. The identification of ten significantly dysregulated mtRNAs between lung cancer tissues and adjacent normal controls, with nine showing significant upregulation and one demonstrating downregulation, aligns with emerging evidence suggesting that mitochondrial dysfunction and altered mitochondrial RNA metabolism represent fundamental characteristics of cancer pathophysiology. These findings are consistent with previous studies by Reznik et al., who demonstrated that mitochondrial respiratory gene expression is frequently suppressed across multiple cancer types (33), suggesting that disrupted mitochondrial RNA processing and metabolism may contribute to the metabolic reprogramming characteristic of malignant transformation.

The superior performance of Random Forest and Logistic Regression algorithms compared to Support Vector Machines in our classification analysis highlights the importance of algorithm selection in biomarker discovery applications. The Random Forest algorithm’s ability to handle high-dimensional data with complex feature interactions while providing interpretable variable importance measures made it particularly well-suited for identifying the most discriminative mtRNA features. The identification of t00043332 as the most significant contributor to cancer classification, derived from mitochondrial tRNA-Tyrosine, suggests that specific mitochondrial tRNA processing pathways may be preferentially disrupted in lung cancer. This finding is supported by recent research demonstrating that stress-induced cleavage of cytoplasmic tRNAs generates functional small RNAs that regulate cellular stress responses and metabolic adaptation (34), suggesting that similar mechanisms may operate in mitochondrial compartments during cancer development.

The absence of significant associations between mtRNA expression levels and overall survival outcomes, despite their excellent diagnostic performance, suggests that these biomarkers may be more valuable for early detection rather than prognostic stratification. This observation is consistent with the concept that different molecular mechanisms may drive cancer initiation versus progression and metastasis. The diagnostic utility of mtRNAs may reflect their involvement in early metabolic reprogramming events that occur during malignant transformation, while survival outcomes may be more influenced by later genetic and epigenetic alterations that drive tumor progression and therapeutic resistance (35). Similar patterns have been observed with other biomarker classes, where diagnostic and prognostic biomarkers often represent distinct molecular signatures reflecting different aspects of cancer biology.

Our functional validation studies demonstrating that t00043332 overexpression promotes proliferation, migration, invasion, and apoptosis resistance in lung cancer cell lines provide compelling evidence for the biological significance of mtRNAs in cancer pathogenesis. The consistent oncogenic effects observed across both A549 and PC9 cell lines, which represent different molecular subtypes of lung adenocarcinoma with distinct genetic backgrounds, suggest that mtRNA-mediated effects may represent a broadly relevant mechanism in lung cancer biology. The promotion of cell invasion and migration capabilities is particularly significant given the critical role of metastasis in lung cancer mortality. These findings are consistent with recent studies by Larriba et al., who demonstrated that mitochondrial small non-coding RNAs exhibit distinct expression patterns during different stages of germ cell development (36), suggesting that mtRNAs may function as important regulators of cellular differentiation and transformation processes.

The apoptosis resistance phenotype conferred by t00043332 overexpression represents a particularly important finding given the central role of apoptosis evasion in cancer development and therapeutic resistance. Mitochondria serve as critical regulators of intrinsic apoptotic pathways through cytochrome c release and caspase activation, and disruption of normal mitochondrial RNA metabolism may contribute to the apoptosis resistance characteristic of cancer cells (37). This observation aligns with previous research demonstrating that mitochondrial dysfunction can promote cancer cell survival through multiple mechanisms including altered calcium homeostasis, modified redox signaling, and disrupted apoptotic machinery (38, 39). The ability of mtRNAs to modulate these processes suggests that they may represent novel therapeutic targets for overcoming apoptosis resistance in lung cancer treatment.

The ratio-based normalization methodology employed in our study addresses a critical challenge in sncRNA biomarker research, where traditional normalization approaches often fail due to the absence of reliable reference genes and the susceptibility of small RNAs to technical variability (40). The success of this approach, as demonstrated by Yu et al. in their validation of mtRNA biomarkers in peripheral blood samples (17), suggests that ratio-based methods may be broadly applicable to other sncRNA classes and cancer types. This methodology is particularly valuable for clinical translation, as it reduces the impact of technical variables such as sample processing conditions, RNA extraction efficiency, and PCR amplification variability that can significantly affect biomarker reproducibility in clinical settings.

The integration of both tissue-based biomarker discovery and functional validation approaches in our study represents an important strength that enhances the clinical relevance of our findings. While many biomarker studies focus exclusively on statistical associations without experimental validation, our demonstration that identified mtRNAs exhibit functional oncogenic properties provides strong evidence for their biological significance and potential therapeutic relevance. This integrated approach aligns with current trends in precision medicine research that emphasize the importance of understanding the mechanistic basis of biomarker associations to improve their clinical utility and identify potential therapeutic targets.

Several limitations of our study warrant consideration in interpreting these findings and planning future investigations. The retrospective nature of the TCGA dataset analysis, while providing access to large sample sizes with comprehensive clinical annotation, may not fully capture the technical variability and pre-analytical factors that influence biomarker performance in prospective clinical settings. Additionally, the focus on two major lung cancer subtypes, while representing the most common forms of the disease, may not encompass the full spectrum of lung cancer heterogeneity, particularly rare subtypes and emerging molecular classifications. Future studies should incorporate prospective validation cohorts with standardized sample collection protocols and expand the analysis to include additional lung cancer subtypes and stages.

The mechanistic understanding of mtRNA function remains limited, and additional research is needed to elucidate the specific molecular pathways through which these small RNAs regulate cellular processes. While our functional studies demonstrate clear oncogenic effects of t00043332 overexpression, the identification of direct target genes and downstream signaling pathways will be essential for understanding the therapeutic potential of mtRNA modulation. Based on its derivation from mitochondrial tRNA-Tyrosine and the observed phenotypic effects, we hypothesize that t00043332 may regulate cellular metabolism through interaction with cytoplasmic RNA-binding proteins or by modulating mitochondrial-nuclear communication pathways. Potential mechanisms may involve stress response pathways and metabolic reprogramming characteristic of cancer cells. Furthermore, the development of effective delivery systems for therapeutic targeting of mtRNAs will require additional research into mitochondrial-specific drug delivery approaches and the pharmacokinetic properties of mitochondria-targeted therapeutics. While traditional miRNA target prediction tools are not directly applicable to mtRNAs due to their mitochondrial origin and distinct biogenesis pathways, emerging approaches including structural similarity analysis and pathway enrichment methods may be adapted for mtRNA functional prediction in future studies. The development of mtRNA-specific computational tools represents an important avenue for advancing our understanding of these regulatory molecules and their potential therapeutic applications.

In conclusion, our study demonstrates that mitochondrial non-coding RNAs represent a promising new class of diagnostic biomarkers and therapeutic targets in lung cancer, with the potential to improve early detection and treatment outcomes. The successful integration of traditional bioinformatics approaches with machine learning methodologies highlights the value of computational approaches in biomarker discovery and validates the importance of combining multiple analytical strategies to advance cancer research. The functional significance of identified mtRNAs, particularly their roles in promoting oncogenic phenotypes, suggests that targeting mitochondrial RNA metabolism may represent a novel therapeutic strategy worthy of further investigation. These findings contribute to the growing understanding of mitochondrial dysfunction in cancer and provide a foundation for future research into mtRNA-based diagnostic and therapeutic applications across multiple cancer types.

## Data Availability

The original contributions presented in the study are included in the article/**Supplementary Material**. Further inquiries can be directed to the corresponding authors.

## Supplementary material

Supplementary Table 1 Clinical and Demographic Characteristics of Study Participants.

Supplementary Table 2 Comprehensive differential expression analysis of mitochondrial non-coding RNAs in lung cancer.
